# Supplementary material for: A Systems Biology Approach to Investigating the Interaction between Serotonin Synthesis by Tryptophan Hydroxylase and the Metabolic Homeostasis
Source: Int J Mol Sci. 2021 Feb 28;22(5):2452. doi: 10.3390/ijms22052452 (PMC7957782; doi:10.3390/ijms22052452)

**Supplementary Figure S1.** TPH1 and TPH2 mRNA expression in colon, adipose tissue, brain and small intestine.


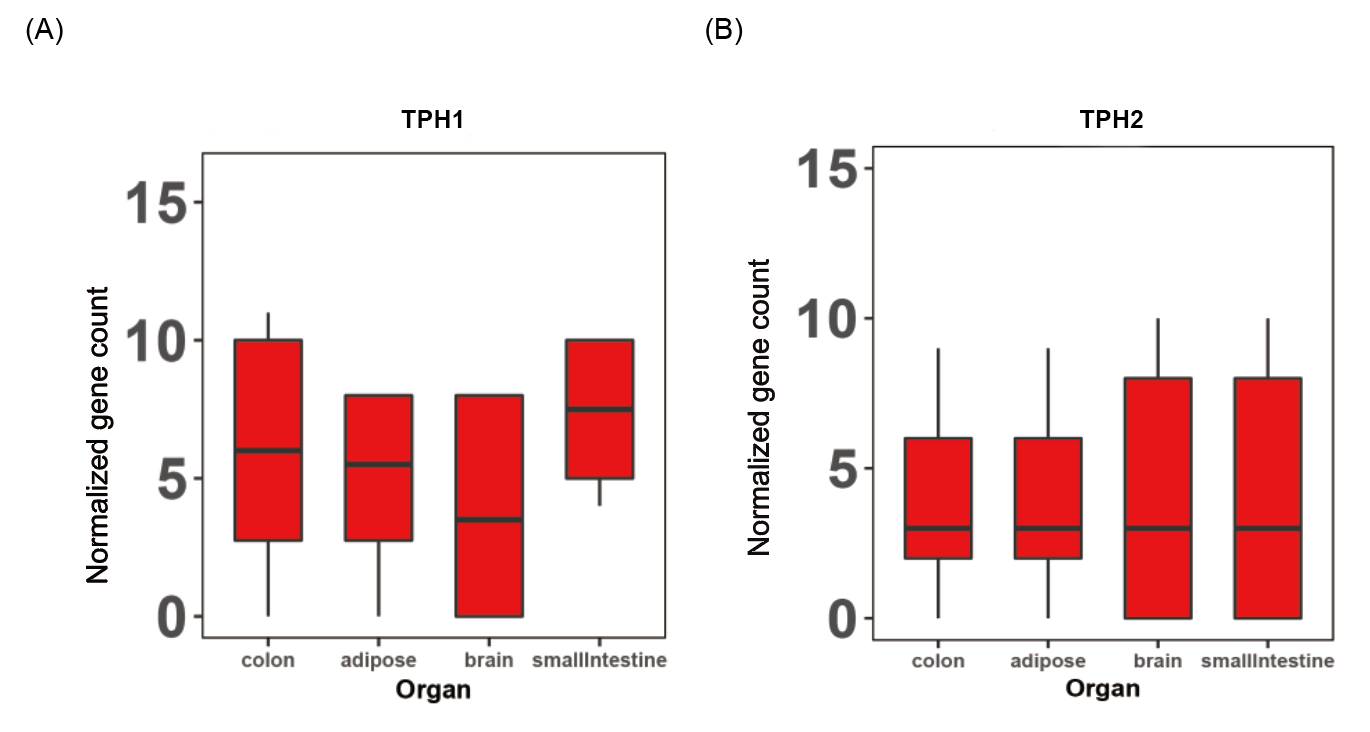


**Supplementary Figure S2.** Comparing TPH1/TPH2 expressions of tissues among low and high TPH groups.


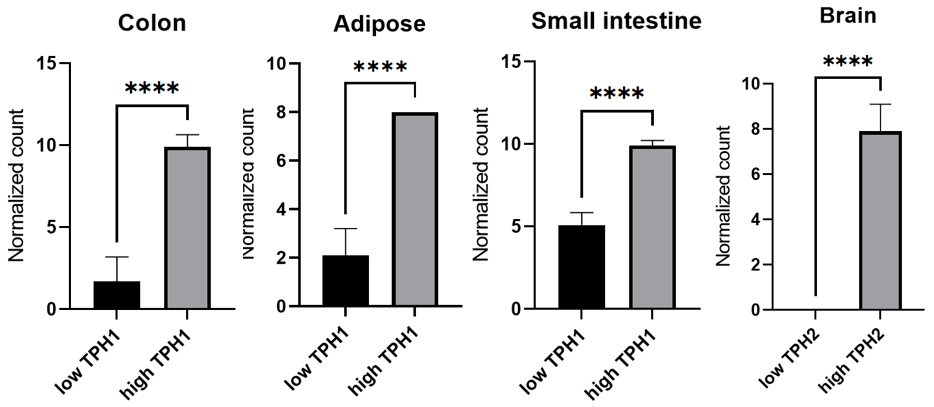


**Supplementary Figure S3.** Gene expression changes in small intestine according to TPH1 expression (n=10 per group). (A) Volcano plot of the significantly differentially expressed genes (DEGs). (B) Heatmap of DEGs. (C-E) DEGs were analyzed by Gene set enrichment analysis. (C) The enrichment plot for pancreatic beta cells. (D) Bar plot depicting the normalized enrichment scores (NES). (E) Dot plot for enriched gene ontology pathways from GSEA results.


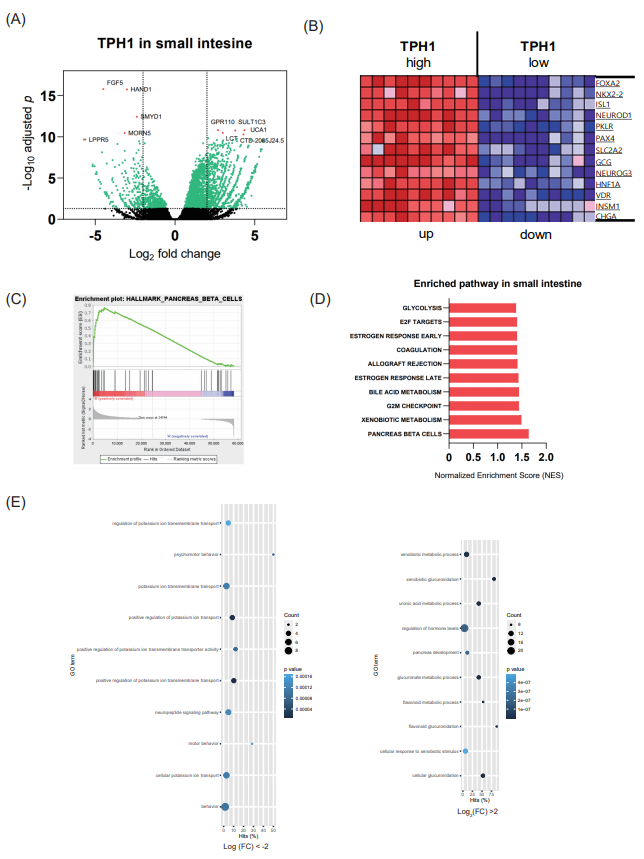

Supplement: Supplementary file 1 [file ijms-22-02452-s001.zip › ijms-1098526 suppl/Supplement figure for IJMS.docx]
